# Complex cognitive and motivational deficits precede motor dysfunction in the zQ175 (190 CAG repeat) Huntington’s disease model

**DOI:** 10.1016/j.expneurol.2025.115350

**Published:** 2025-06-23

**Authors:** D.J. Harrison, P. Linehan, Y. Patel, Z. Bayram-Weston, A.E. Rosser, S.B. Dunnett, S.P. Brooks, M.J. Lelos

**Affiliations:** a Brain Repair Group, School of Biosciences, Cardiff University, Cardiff CF10 3AX, UK; b Medicines Discovery Institute, Cardiff University, Cardiff CF10 3AX, UK

**Keywords:** Huntington’s disease, zQ175, Mouse model, Cognition, Neuropsychiatric, Motor, Behaviour

## Abstract

Huntington’s disease (HD) is a progressive, inherited neurodegenerative disorder characterised by motor, cognitive, and neuropsychiatric dysfunction for which several mouse models have been developed. Knock-in models, such as zQ175, retain the genetic context observed in people with HD by introducing CAG repeats into the native huntingtin gene. In this study, we conducted a comprehensive, longitudinal analysis of phenotypic changes in the zQ175 mouse, with a focus on exploring the emergence of complex cognitive processes. Our findings indicate that robust cognitive and motivational deficits precede motor dysfunction in this model, with some apparent sex differences. Specifically, male zQ175 mice were slower to habituate to a novel environment and they showed impaired sensorimotor gating, in comparison to female mice. By 12 weeks old, cognitive deficits were observed in zQ175 mice of both sexes on a Pavlovian classical conditioning task. Reduced motivation to work for reward was identified as early as 27 weeks, while attentional and visuospatial deficits were also detected in the 5-choice serial reaction time task. Implicit learning deficits were identified at 30 weeks. zQ175 mice were hypoactive at ~24 weeks but became hyperactive by 60 weeks of age. Motor impairments emerged by 24 weeks for females and 48 weeks for males. Thus, we observed a wide range of cognitive deficits (attentional, visuospatial, sensorimotor, instrumental and implicit learning), as well as a gradual progression of motor changes. This detailed phenotypic timeline establishes the face validity of this model insofar as these mice present with complex neuropsychiatric and cognitive impairments that are evident in people with HD.

## 1 Introduction

Huntington’s disease (HD) is a progressive inherited neurodegenerative disease caused by the expansion of the polyglutamine (CAG) repeat in the N terminal of the huntingtin gene (*HTT*). People with HD experience progressive motor dysfunction, often preceded by cognitive impairment and neuropsychiatric disturbances (Snowden, 2017). Prodromal cognitive changes are evident, with function continuing to decline throughout the course of the disease. These changes include deficits in attention, object and space perception, executive function, episodic memory, processing speed and visuospatial attention. The PREDICT-HD study followed premanifest participants carrying the HD mutation, grouped according to an estimate of their probability of developing motor symptoms within 5 years (low probability: >12 years until predicted onset; medium probability: 7.59–12.78 years; and high probability: <7.59 years). Cognition was reported to decline over time on all measures (Paulsen et al., 2014) and different deficits emerged as the disease progressed for each group. Patients in the low probability group exhibited cognitive deficits in fewer measures than those in medium and high probability groups, who exhibited successively more cognitive signs as motor onset probability increased (Kirkwood et al., 1999). Behavioural and neuropsychiatric symptoms such as apathy, anxiety, obsessive behaviour and psychosis, also present differently according to disease stage. The PREDICT-HD study found significant changes over time in premanifest participants compared to controls in eight out of nine psychiatric measures (Paulsen et al., 2014), including apathy, a core neuropsychiatric feature of HD with an early manifestation (Teixeira et al., 2016). Interestingly, many of the clinical markers of disease progression (e.g., cognitive and psychiatric variables) were found to progress in a relatively linear fashion and to decline in close correlation with biological markers of brain imaging changes. Motor and functional variables, on the other hand, progressed in a nonlinear fashion, which is reflected by the fact that motor signs and functional impairment became evident only at certain points of disease progression. Disease signs can also vary according to sex in manifest HD, with males performing better in cognitive tests than females (Hentosh et al., 2021). Females present with more severe depressive symptoms than males; however, both sexes progressed with a similar trajectory of decline (Hentosh et al., 2021). No differences were reported for irritability or psychosis (Hentosh et al., 2021).

Since the discovery of the disease-causing genetic mutation, several animal models have been generated to advance our understanding of HD and aid in the development of novel treatment options. The zQ175 knock-in (KI) mouse model recapitulates the genetic context seen in people with HD, since the extended polyglutamine repeat construct is inserted within the native murine huntingtin gene (*Htt*). The zQ175 mouse model originated from the CAG 140 model due to spontaneous intergenerational CAG expansion, which has continued so that the CAG repeat length is approximately 191 at the time of writing. Previously, cognitive changes in heterozygous zQ175 mice were identified from 4 months (Shobe et al., 2021) and motor deficits from 8 months of age (Heikkinen et al., 2012; L. B. Menalled et al., 2012). In addition, zQ175 mice show other early gene expression changes known to be altered in HD, such as dopamine- and cAMP-regulated phosphoprotein 32 kDa (*Darpp-32*) and neuronal mutant huntingtin (mHTT) aggregates from as early as 6 weeks of age in the striatum (L. B. Menalled et al., 2012; Smith et al., 2023), suggesting that behavioural changes may manifest earlier than previously detected. While previous research has demonstrated that zQ175 mice present with some phenotypes seen in HD, a more rigorous and detailed characterisation is needed to determine the specific deficits and their timeline. The impact of sex within this strain remains poorly understood and a greater appreciation of the prevalence of cognitive and neuropsychiatric symptoms in HD patients means that a detailed characterisation of these psychiatric disease features is critical for the effective use of this model in future research.

This study aimed to establish a timeline of cognitive, neuropsychiatric and motor behavioural changes in zQ175 mice to better characterise this model for preclinical use. We assessed both male and female zQ175 mice from 12 weeks to 84 weeks of age. Where studies were sufficiently well powered, the effect of sex was analysed. The phenotypic timeline presented in this study will aid researchers in effectively utilizing the zQ175 model for the analysis of novel treatments.

## 2 Methods

### 2.1 Mice and cohorts

Five female and five male heterozygous B6J.129S1-Htttm1Mfc/190JChdi (zQ175) mice were obtained from The Jackson Laboratory (Bar Harbor, Maine, USA; Strain #:370476) and bred to the C57BL6/J background to produce the wildtype (WT) and heterozygous zQ175 male and female offspring used in this study. Genotyping and CAG repeat length of individuals was determined from tail tissue biopsies by Laragen (Culver City, CA, USA). The CAG repeat length of this behavioural cohort was 190 ± 1 (mean ± SEM). Mice in cohorts 1, 2 and 3 were bred as part of the same generation (ensuring no generational CAG repeat expansion effects) and tested concurrently.

Behavioural test batteries were conducted within three cohorts of animals, each undergoing a series of longitudinal tests:

1. Cohort 1: Rotarod > grip strength > locomotor activity > pre-pulse inhibition > balance beam at 12, 24, 36, 48 and 60 weeks of age. *n* = 20 male WT; 20 male zQ175; 11 female WT and 20 female zQ175.
2. Cohort 2: Pavlovian classical conditioning > progressive ratio > 5-choice serial reaction time task > serial implicit learning task > open field > sucrose preference at 12 to 27, 48 to 66 and 78 to 82 weeks of age. *n* = 7 WT (4 male 3 and female); 8 zQ175 (4 male and 4 female).
3. Cohort 3: Pavlovian classical conditioning > delayed alternation at 12 to 24 and 48 weeks of age. *n* = 8 WT (4 male and 4 female); 8 zQ175 (4 male and 4 female).

Experimental animals were housed in an environmentally controlled holding room maintained at a temperature of 21 ± 2 °C and 50 ± 10 % humidity under a 12-h light-dark cycle. Mice were housed in mixed-genotype sex-matched cages of four wherever possible. All cages contained woodchip bedding, wooden chew-stick, cardboard tube and nestlet bedding material and were cleaned weekly after daily behavioural testing was complete. Standard lab chow (SDS RM1E, Special Diet Services) and tap water were available ad libitum, unless under water restriction protocols described below.

### 2.2 Behavioural testing

Methods for behavioural testing have been described in detail else where (Brooks et al., 2012a; Brooks et al., 2012b; Brooks et al., 2012c), but brief descriptions are provided below. Researchers were blinded to the genotype and sex of the mice during behavioural testing and analysis.

### 2.3 Body weight

Mice were weighed weekly, except for when on a water-restricted schedule (operant testing) during which time they were weighed every 2 days in accordance with the Project License.

### 2.4 Rotarod

Mice were trained on the accelerating rotating beam (3 cm Ø) of the rotarod (Ugo Basille) for 5 mins daily for 5 days. Each time a mouse fell, they were immediately returned to the beam until the full time had elapsed. On the day of testing, mice were given a single reminder run followed 5 min later by two test runs separated by a 5-min rest period. The latency to first fall from the rod was recorded and the mean of the two test runs used for analysis.

### 2.5 Grip strength

Mice were placed on a 30 × 30 cm wire mesh consisting of a grid of 1 × 1 cm squares. The grid was inverted slowly through the horizontal plane approximately 15 cm above a double folded towel on the bench surface and held in position for a maximum of 60 s. The time to lose their grip and fall to the towel was recorded as a measure of grip strength.

### 2.6 Locomotor activity

16 Perspex chambers (Med Associates, St. Albans, VT, USA, 42 × 26 × 19 cm) intersected by 3 infra-red beams spaced 10 cm apart were used to measure locomotor activity. The number of non-perseverative beam breaks made was considered as the indicator of activity level. At each testing point the mice were placed in the chamber for 27 h with free access to food and water. Activity over the initial 30 min was measured as habituation, and movement over a subsequent 24-h period, split between light and dark phases, was measured to capture activity changes within a single circadian cycle.

### 2.7 Acoustic startle and pre-pulse inhibition (PPI)

Animals were placed in the restraining tubes of the startle chambers (San Diego Instruments, San Diego) and exposed to a 5 min white noise (70 dB) habituation period that remained over the duration of the 45-min test period. Presentation of 120 dB acoustic startle stimuli of 50 ms were paired with a pre-pulse stimulus of 20 ms at 16 dBs over background white noise. A constant interval of 10 ms between pre-pulse and startle stimuli was set, with a pseudorandom inter-trial interval (ITI) duration between 5 and 30 s. Trials in which no pre-pulse stimulus was presented were used to assess baseline levels of startle response.

### 2.8 Balance beam

Mice were trained to traverse an inclined (17° from horizontal) 100 cm wooden beam to a dark, enclosed goal box. The beam tapered in width from 120 mm at the base, to 5 mm at the goal box. The goal box represents a safe refuge in this test which motivates the mice to seek it. Training consisted of habituating the mice to the goal box for 1 min, followed by placement on the beam at ever increasing distances from the goal box, allowing mice to run up the beam and enter the box for 1 min before being removed. Mice were subsequently recorded performing two test trials 24 h later. For each test trial mice were first placed on the lower end of the beam facing away from the goal box. The time taken to turn around and face the incline was recorded as “time to turn”. A maximum of 1 min was permitted after which time the mouse scored 60 s and was physically placed on the beam facing the correct direction. The time taken to traverse the beam was recorded (between the ‘start’ line 10 cm from the wide low end, 80 cm to the finish line, 10 cm from the goal box. Complete traversal was interpreted as all four limbs crossing over the finish line). During the running of the beam, fore- and hind-limb foot slips were live-counted from one side of the beam and recorded on the other for later analysis. A 15 mm wide ledge 2 cm below the top surface of the beam allowed mice to slip without falling. The mice were given 2 min to traverse the beam with failure incurring a time of 120 s. Mice were permitted 1 min in the goal box prior to starting the next trial. The mean measures from both trials were analysed and compared.

### 2.9 Operant training and testing

Operant training procedures have been described in detail elsewhere (Harrison et al., 2021). Briefly, mice were gradually water restricted over the course of 1 week from ad lib to 3 h free access per day. During this period mice were exposed to the operant reward (Yazoo Strawberry Milk, Campino, UK) in home cages to prevent neophobic response behaviours in the testing period. Cognitive testing was conducted in 14 × 13.5 × 13.5 cm operant chambers within sound-attenuated boxes controlled by a BehaviourNet Controller BNC MKII operating system (Campden Instruments, Loughborough, UK). Each chamber consisted of an array of 9 nose-poke holes across the back wall, and a reward magazine on the opposing wall flanked either side by an additional nose-poke hole. Each hole and the magazine contained a bulb to illuminate the hole and an infrared sensor to detect nose entries. A peristaltic pump was set to deliver strawberry milk reward to the magazine. Two house-light bulbs were located at the top of the side wall panels to illuminate the chamber.

### 2.10 Pavlovian classical conditioning task (CCT)

Blockades were used to cover all nose-poke holes throughout the CCT. Mice received an initial 20 min session in which they could freely explore the chamber with the house lights illuminated throughout. Mice had free access to a 500 μl reward which was dispensed to the magazine at the beginning of the session. CCT was conducted over 10 consecutive daily sessions of 15 trials. For each trial, a pseudorandom inter-trial interval (ITI) of 60, 75, 90, 105 or 120 s, during which all lights were off, was followed by a 10 s illumination of the houselights (conditioned stimulus (CS)), and delivery of 12.5 μl to the magazine. When the reward was collected, the houselights would be extinguished and a 5-s delay triggered, after which the next ITI would begin. The delay timer was reset if an animal entered the magazine within the 5 s wait period and a perseverative response recorded. For the final 10 s of each ITI period, and the 10 s CS presentation, the number of entries to the magazine was recorded in 2 s time bins.

The difference in the number of responses made in the CS and the final 10 s ITI period was calculated and plotted to assess progress of the CS-reward association using the formula: Total Conditioned Pokes = (CS(n) - ITI(n)), where CS(n) = the total number of magazine entries made during the 10 s CSs (total 150 s) and ITI(n) = the total number of magazine entries made during the final 10s of the ITIs (total 150 s). The slope of the line was taken as the rate of learning and calculated using the formula: m(i) = (r(i) × sy(i))/(sx(i)), where (i) = individual animal data set, m(i) = slope, sy(i) = standard deviation of y values (Total Conditioned Pokes), sx(i) = standard deviation of the x values (session number), and r(i) = correlation between x and y values calculated using the formula: r(i) = (covariance x(i) and y(i))/(sx(i) × sy(i)).

Following the CCT, an extinction test was conducted as a measure of cognitive flexibility, in which the reward pump was disconnected from the magazine (i.e. no reward was delivered). During the extinction session mice learn a new association whereby CS ≠ reward. Extinction data were calculated as CS pokes made during each extinction test trial was compared to the mean number of CS entries made during the final CCT session.

### 2.11 Operant conditioning

On completion of the CCT extinction test, mice were trained to respond in nose-poke holes in a simple fixed-ratio paradigm. Covers were removed from the holes either side of the magazine (L & R). During daily 20 min trials both L & R would be illuminated. A response made in either would trigger the L & R lights to turn off, illumination of the magazine light and delivery of a 50 μl reward. On collection of the reward the magazine light was extinguished and a 2-s ITI initiated during which all lights remained off. Performance was monitored and low-responding mice were encouraged to engage by painting reward at the back of the illuminated holes. Progression to the next phase was made when 50 self-initiated responses per session were complete.

### 2.12 For animals in cohort 2

Following on, mice were trained in a similar fixed-ratio paradigm but with the centre hole of the array (C) illuminating on the back wall, instead of L & R. Criteria for progressing was set to at least 100 responses in two consecutive sessions, or after ten sessions. All mice were run in the final session.

#### 2.12.1 Progressive ratio (PR) task

The PR task probes motivation and requires mice to work harder (increased nose poke/reward ratio) for each successive reward, until the point (or threshold) where the mouse no longer works to obtain the reward. With the test chamber set up as before, the ratio of responses/reward increased within the 90-min session with every 3 repetitions of the current ratio, so as the following sequence of nose pokes is required for each reward: 1, 1, 1, 3, 3, 3, 6, 6, 6, 9, 9, 9 and so on. Outcome measures analysed for PR were the maximum ratio attained, the ratio at which there was a 60 s break in responding, and the latency to reach that 60 s breakpoint.

#### 2.12.2 Five-choice serial reaction time task (5CSRTT)

This task is an analogue of a human task designed to measure visuospatial acuity. During the 5CSRTT, five holes in the back wall array were uncovered (A’, ‘B’, ‘C’, ‘D’ and ‘E’) whilst L & R holes remained covered. An initial 30-min session was entered whereby one of the five holes was pseudo-randomly illuminated per trial (stimulus). A response to the illuminated hole would result in the light switching off, illumination of the magazine and delivery of a 25 μl reward. Following collection of the reward, a 5-s ITI was initiated prior to the next trial. Failure to respond within 30 s, or a response in an incorrect hole resulted in a time out (TO) period in which all lights were extinguished for 5 s before the next trial. In the next five session blocks, the stimulus length was reduced to 10 s, 2 s and finally 0.5 s, with a 10 s limited hold in all sessions in which mice were able to respond. Data were analysed by each mouse in each session for total trials started (TTS), response accuracy (= (Correct responses)/(Correct + Incorrect responses)), mean time taken to make a correct response (CRT), number of time-outs incurred (TO), and number of anticipatory pokes made during the ITI or TO periods.

### 2.12.3 Serial implicit learning task (SILT)

For SILT, mice must make a series of two pokes to gain a reward. Firstly, as with 5CSRTT, a stimulus light (S1) is presented in one of the five nose-poke holes. The S1 remains lit until a response (correct or incorrect) is made. With a successful response to S1 the light is extinguished, and a second light is presented (S2) randomly in one of the 4 alternative holes. A correct response to S2 results in reward delivery to the magazine and a 2 s ITI following reward collection. An incorrect response to either the S1 or S2 stimuli results in a 5 s TO followed by the presentation of a new trial. The implicit learning aspect of the task relates to the identification of a predictable sequence embedded within the randomly presented trials. The predictable trial is always the same; if S1 is “B” then S2 is always “D” (B → D), and performance of this combination is measured against the randomly presented, thus unpredictable, D → B antisense combination. The mice were trained for five sessions with an S2 duration of 2 s, after which this was reduced to 0.5 s for the five test sessions.

### 2.13 For animals in cohort 3

#### 2.13.1 Delayed alternation (DA)

Following operant conditioning, mice from cohort 3 progressed to the DA task, during which L & R holes remained open. Each session started with both L & R holes illuminated. A response in either hole resulted in both lights being extinguished and illumination of the magazine. A response to the magazine started a delay timer pseudo-randomly selected from delay sets of increasing duration per session (0 s; 0, 0.5, 1, 1.5 or 2 s; 0, 1, 2, 3, 4 or 5 s; then 1, 2, 3, 4, 5, 6 s), after which both L & R holes were illuminated again. To obtain the reward the mouse must respond in the hole opposite to its previous response. A correct response resulted in reward delivery and the delay timer was started for the next trial. A lack of response or incorrect response incurred a 2 s TO penalty during which all lights were extinguished before both L & R nose-poke hole illuminated. To progress to each successive delay set, mice must reach a criterion of 85 % accuracy at the lowest delay. After reaching the final delay set, mice were tested for five consecutive days and the mean data used for analyses. Data is presented as percent accuracy (total correct/total incorrect responses x 100) for each delay length.

### 2.13.2 Sucrose preference test

Perceived reward value was tested following completion of rewarded operant testing. Mice were housed individually from 8 am to 4 pm for four consecutive days. A dish containing 4 g sucrose pellets was fixed in the cage and weighed again at 4 pm each day. The mean weight of sucrose consumed across the final three days was used for analyses.

### 2.13.3 Open field

Velocity, as a measure of locomotor function, was measured in an open field arena (80 × 80 cm). After having been habituated for 15 min the day before, mice were placed in the centre of the arena and recorded for 15 min with an overhead camera connected to a PC running Ethovision tracking software (Version 2.3.19, Noldus Information Technology, The Netherlands).

### 2.13.4 Histology and stereology

Subcohort of excess mice from Cohort 1 were transcardially perfused at 12 weeks (*n* = 5× WT, 4× zQ175), 24 weeks (*n*= 6× WT, 5× zQ175), 36 weeks (n = 5× WT, 6× zQ175), 48 weeks (n = 5× WT, 5× zQ175), 60 weeks (n = 6× WT, 6× zQ175) and 72 weeks (*n* = 4× WT, 4× zQ175). Processing of tissue was conducted as previously reported (Bayram-Weston et al., 2016a). Mice were anaesthetized by intraperitoneal injection of 0.2 ml of Euthetal (Merial, Essex, UK) and perfused intracardially with phosphate-buffered saline (PBS, pH 7.4) for 3 min and 4 % paraformaldehyde (PFA) (Fisher Scientific, Loughborough, UK) in a 0.1 M PBS solution, pH 7.4, for a further 5 min. Brains were carefully removed, post fixed in 4 % PFA for 4 h, and transferred to 25 % sucrose in PBS for 24 h or until they sunk to the bottom of the container. Brains were cut in 40 μm coronal series of 1:12 using a freezing sledge microtome (Leitz Bright Series 8000, Germany) and cryoprotected by immersion in antifreeze solution. Prior to immunohistochemistry, tissue sections were placed in (pH 7.4) TRIS Buffered Saline (TBS), and washed twice for 5 min. Sections were pre-treated with an antigen retrieval method by incubation in citrate buffer (pH 6) for 20 min at 95 °C. Endogenous peroxidise activity was inhibited by incubation in methanol containing 3 % H_2_O_2_ (VWR International, UK) for 5 min. Non-specific binding sites were blocked with 3 % horse serum in TBS for 1 h, and sections incubated with S830 (1:20000 with 1 % horse serum) overnight (~16 h) at room temperature (21° ± 2 °C). After several washes in TBS, sections were incubated with a horse anti-goat secondary antibody (diluted 1:200, Vector Laboratories, Burlingame, CA, USA) for 2 h. After several washes in TBS, sections were incubated with a biotin-streptavidin kit according to the manufacturer’s instructions (Vector Laboratories)then rinsed in TBS. The peroxidase activity was visualized with 3,3′-diaminobenzidine (DAB: Sigma-Aldrich, Poole, UK), and the sections mounted on gelatine-coated slides prior to dehydration and cover-slipping.

For cresyl violet staining a 1:12 series of sections were mounted onto slides and allowed to air dry before serial incubation for 5 min each in: 70 % IMS, 95 % IMS, 100 % IMS, 50/50 chloroform alcohol, 95 % IMS, 70 % IMS, distilled water, cresyl violet solution and distilled water. Sections were dehydrated in 70 % and 95 % IMS, then destained by agitation in acid alcohol solution and cover-slipped using DPX mountant.

Two-dimensional stereology was performed on an Olympus BX50 microscope (Olympus Optical Co. Tokyo, Japan) with PC-based image analysis software (Olympus C.A.S.T. grid system v1.6.). Cell counts were performed on a 1:6 series of S830-stained sections throughout the entire left striatum of each mouse. Briefly, the striatum was outlined under a 4× objective lens and the enclosed area was calculated by the C.A.S.T grid software. Sections within the defined volume of the striatum were sampled at random and cells counted under a 100× objective lens using a 265 μm^2^ 2D optical dissector counting frame. The total number of affected cells in the structure per section was calculated using the Abercrombie correction:

$$C=\sum c\times (\frac{\sum A}{\sum (a\times n)})\times f\times (\frac{M}{M+D})$$

Where *C* = estimate for the total number of cells; *c* = number of cells counted in each sampling frame; *A* = area of striatum for each section; *a* = area of sampling frame; *f* = sectioning frequency; *M* = section thickness and *D* = mean cell diameter.

Cells were counted and categorised in terms of expressing either diffuse nuclear staining alone or containing frank nuclear inclusions (NIIs) with or without additional diffuse nuclear staining.

#### 2.13.5 Statistics

All data were analysed using IBM SPSS software (version 29). Data were split by sex for Cohort 1, which was sufficiently well powered to consider the performance in each sex. For Cohorts 2 and 3, data from male and female mice were pooled due a lower ‘n’ in each group. Data generated were analysed by either repeated measures 2-way ANOVA with factors Genotype x Age for body weight, rotarod, grip strength, locomotor activity, pre-pulse inhibition and balance beam, classical conditioning (responses by session, extinction test), progressive ratio, sucrose consumption and open field; factors Genotype x Stim, 5CSRTT; or factors Genotype x Paradigm for SILT; by repeated measures 3-way ANOVA with factors Genotype x Time x CS for classical conditioning (response rate within trial) or factors Genotype x Age x Delay for delayed alternation); or by student’s *t*-test for classical conditioning (learning slope, responses per session, perseverative responses); or by non-repeated measures two-way ANOVA with factors of Genotype x Age for striatal volume, glial and neuronal cell counts; or by one-way ANOVA for aggregate staining. Body weight was included as a covariate for rotarod, grip strength and balance beam. Where appropriate, simple main effects were tested using the Šidák correction for multiple comparisons. For one-way ANOVA post hoc comparisons Tukey correction was used. Any missing values were estimated using linear interpolation. Missing values were replaced when animals had prematurely died or when operant apparatus had temporarily malfunctioned to allow for repeated measures analysis. An average of 4 missing values were estimated for any variable with missing values. Data are presented as mean and standard error of the mean (SEM).

## 3 Results

Wildtype (WT) and zQ175 heterozygous mice of both sexes underwent behavioural testing to probe longitudinal cognitive and motor function. Subjects were tested in three cohorts. The first cohort underwent a broadly motor test battery, with data compared for genotype and sex effects. The second and third concentrated more on cognitive function, with male and female data pooled for analyses due to smaller group sizes.

### 3.1 Body weight

**Fig. 1:**
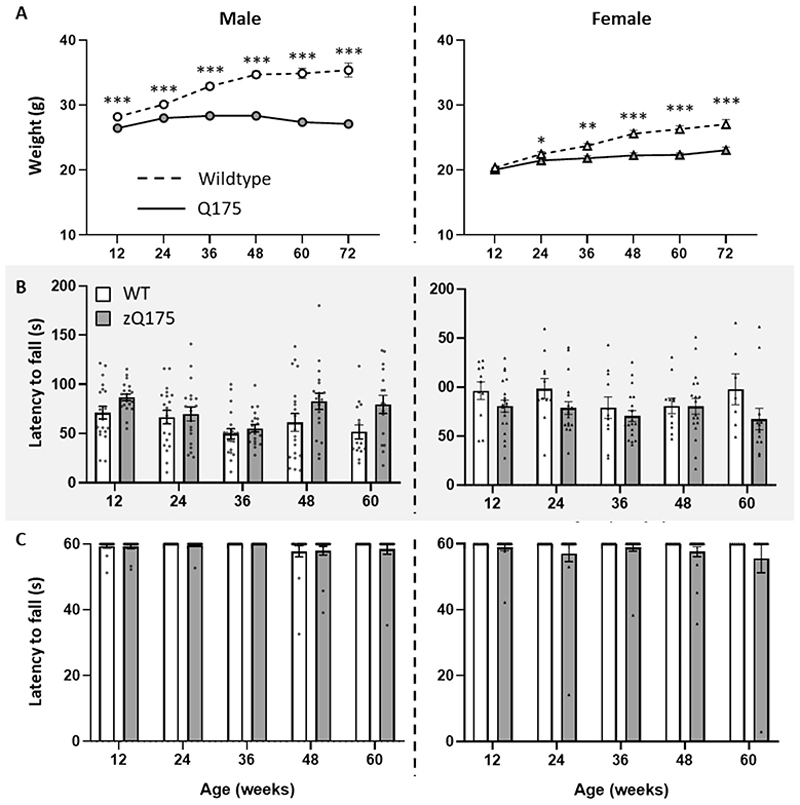
A) Body weight. Mean body weights for male and female mice (left and right panels respectively) from 12 to 72 weeks of age. B) Rotarod. Mean latency to fall from the accelerating rotarod for male and female mice (left and right panels respectively) from 12 to 60 weeks of age. C) Grip strength. Mean latency to fall for male and female mice (left and right panels respectively) from 12 to 60 weeks of age. Data are presented as group means ± SEM; Pairwise comparisons at each age: * *p* < .05, ** *p* < .01, *** *p* < .001.

Both male and female zQ175 weighed significantly less than their WT littermates (Fig. 1A, Genotype: F_1,38_ = 144.94 and F_1,29_ = 30.79 respectively, both *p* < .001). The zQ175 males were lighter from 12 weeks of age (Genotype x Age: F_5,34_ = 47.54, *p* ≤ 0.001; pairwise comparisons *p* < .001 at all ages). Female weights did not differ until 24 weeks (Genotype x Age F_5,25_ = 10.93, *p* ≤0.001; pairwise comparisons 12w, *n.s*.; 24w, *p* < .05; 36w, *p* < .01; thereafter, all *p* < .001).

### 3.2 Rotarod

In the rotarod test of motor coordination, no difference in latency to fall from the rotating beam was observed between either zQ175 and WT males or females when analysed with weight as a covariate (Fig. 1B, Genotype: F_1,37_ = 0.00 and F_1,28_ = 1.02 respectively, both *n.s*.).

### 3.3 Grip strength

No significant differences in grip strength, as measured by time to fall from an inverted grid, were observed between zQ175 and WT males or females (Fig. 1C, Genotype: F_1,37_ = 1.30 and F_1,29_ = 1.28, respectively, both *n.s*.).

### 3.5 Locomotor activity

**Fig. 2:**
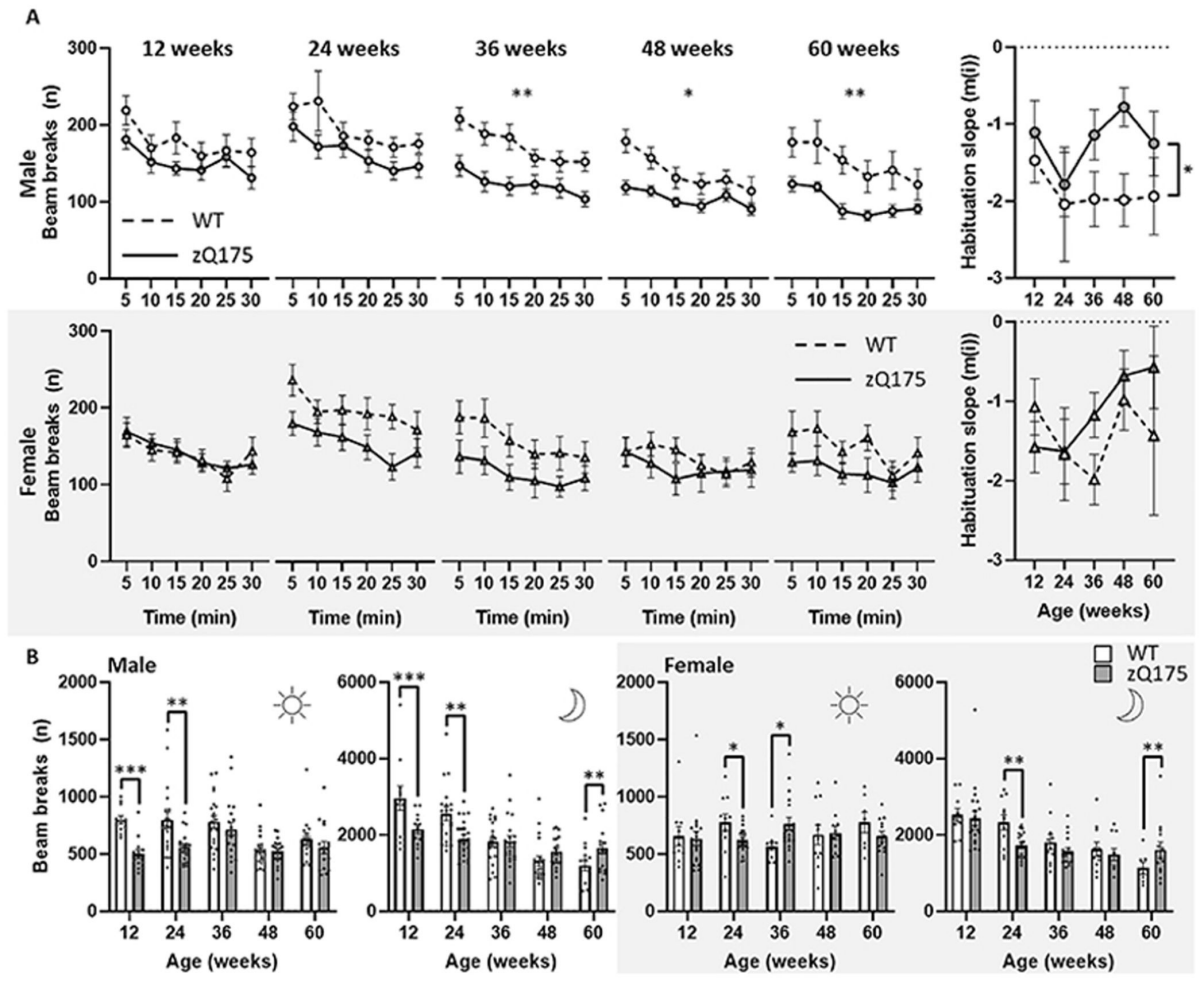
A) Locomotor activity habituation. Mean number of beam breaks made per 5 mins during a 30 min habituation period for male and female mice (top and bottom rows respectively) from 12 to 60 weeks of age (left panels) and rate of habituation at each age (right panels). B) 24-h locomotor activity. Mean number of beam breaks made during the light phase (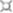, 6 am to 6 pm) and dark phase (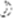, 6 pm to 6 am) by male and female mice (left and right panels respectively). Data are presented as group means ± SEM; Pairwise comparisons at each age: * *p* < .05, ** *p* < .01, *** *p* < .001.

When habituating to locomotor activity arenas (initial 30 mins) male zQ175 exhibited reduced exploratory behaviour, making fewer infra-red beam breaks than WT from 36 weeks old (Fig. 2A, Genotype: 12w: F_1,23_ = 2.14 and 24w: F_1,37_ = 2.57, both *n.s*.; 36w: F_1,38_ = 10.27, *p* < .01; 48w: F_1,38_ = 6.55, *p* < .05 and 60w: F_1,26_ = 8.62, *p* < .01). The rate of habituation in the first 30 mins was slower for zQ175 than WT males (Habituation Slope. Genotype: F_1,38_ = 5.77, *p* < .05). In contrast, females showed no significant differences in movement in the habituation period (Genotype: 12w: F_1,27_ = 0.01; 24w: F_1,28_ = 3.50; 36w: F_1,28_ = 2.63; 48w: F_1,21_ = 0.30 and 60w: F_1,18_ = 1.64, all *n.s*.) or rate of habituation (Habituation Slope. Genotype: F_1,29_ = 0.23, *n.s*.). During the light phase of locomotor activity tracking (6 am – 6 pm) male zQ175 were less active than WT at both 12 and 24 weeks, but no significant difference was observed from 36 weeks onward (Fig. 2B **panel 1**, Genotype x Age: F_4,152_ = 2.90, *p* < .05; pairwise comparisons 12w, *p* < .001; 24w, *p* < .01; thereafter, all *n.s*.). Similarly, during the dark phase, male zQ175 were less active than WT at 12 and 24 weeks, but by 60 weeks were more active (Fig. 2B **panel 2**, Genotype x Age: F_4,152_ = 11.76, *p* < .001; pairwise comparisons 12w, *p* .001; 24w, *p* < .01; 36w and 48w, both *n. s*. and 60w, *p* < .01). For female mice, zQ175 were less active in the light phase than WT at 24 weeks, but more active at 36 weeks, with no sig-nificant difference in locomotor activity at other time points (Fig. 2B **panel 3**, Genotype x Age: F_4,116_ = 3.75, *p* < .01; pairwise comparisons 12w, *n.s*.; 24w and 36w, both *p* < .05; thereafter, all *n.s*.). In a similar pattern to males, zQ175 females in the dark phase were less active than WT at 24 weeks but more active at 60 weeks (Fig. 2B **panel 4**, Genotype x Age: F_4,116_ = 5.01, *p* < .001; pairwise comparisons 12w, *n.s*.; 24w, *p* < .01; 36w and 48w, both *n.s*. and 60w, *p* < .01). This could suggest the movement deficits observed in male zQ175 during habituation were not driven by an overt motor difference but may be indicative of reduced motivation to explore the novel environment, differences in anxiety and/or differences in recognizing repeated exposure to the activity box environment over time.

### 3.5 Pre-pulse inhibition

**Fig. 3:**
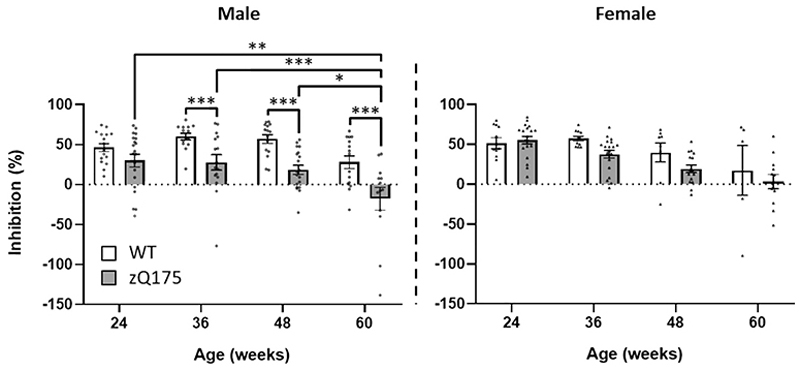
Acoustic startle and pre-pulse inhibition. Percentage reduction from baseline response to a 120 dB startle stimulus after a 16 dB pre-pulse for male and female mice (left and right panels respectively) from 12 to 60 weeks of age. Data are presented as group means ± SEM; Pairwise comparisons: * *p* < .05, ** *p* < .01, *** *p* < .001.

The pre-pulse inhibition test measures sensorimotor gating, which is the ability of the nervous system to filter out or inhibit irrelevant sensory information. Specifically, pre-pulse inhibition assesses how well an individual can suppress its startle response when a weaker stimulus (pre-pulse) precedes a stronger startling stimulus. Given a 16 dB pre-pulse indicator, male zQ175 were less able to inhibit their startle response to a 120 dB click compared to WT (Fig. 3A, Genotype: F_1,38_ = 41.80, *p* < .001). This deficit was evident in zQ175 males from 36 weeks onwards (Genotype x Time: F_3,114_ = 2.72, *p* < .05; pairwise comparisons 24w, *n. s*.; thereafter, all *p* < .001). Over the entire testing period, whilst the ability of zQ175 males to inhibit startle response declined over time, WT performance did not (pairwise comparisons WT 24w vs 60w, *n.s*. and zQ175 24w vs 60w, *p* < .01). Under the same conditions no significant differences were seen between females (Genotype: F_1,29_ = 2.32, *n.s*.), with both zQ175 and WT response declining with time (Time: F_3,87_ = 13.57, *p* < .001; pairwise comparisons WT 24w vs 60w, *p* < .05 and zQ175 24w vs 60w, *p* < .001).

### 3.6 Balance beam

**Fig. 4:**
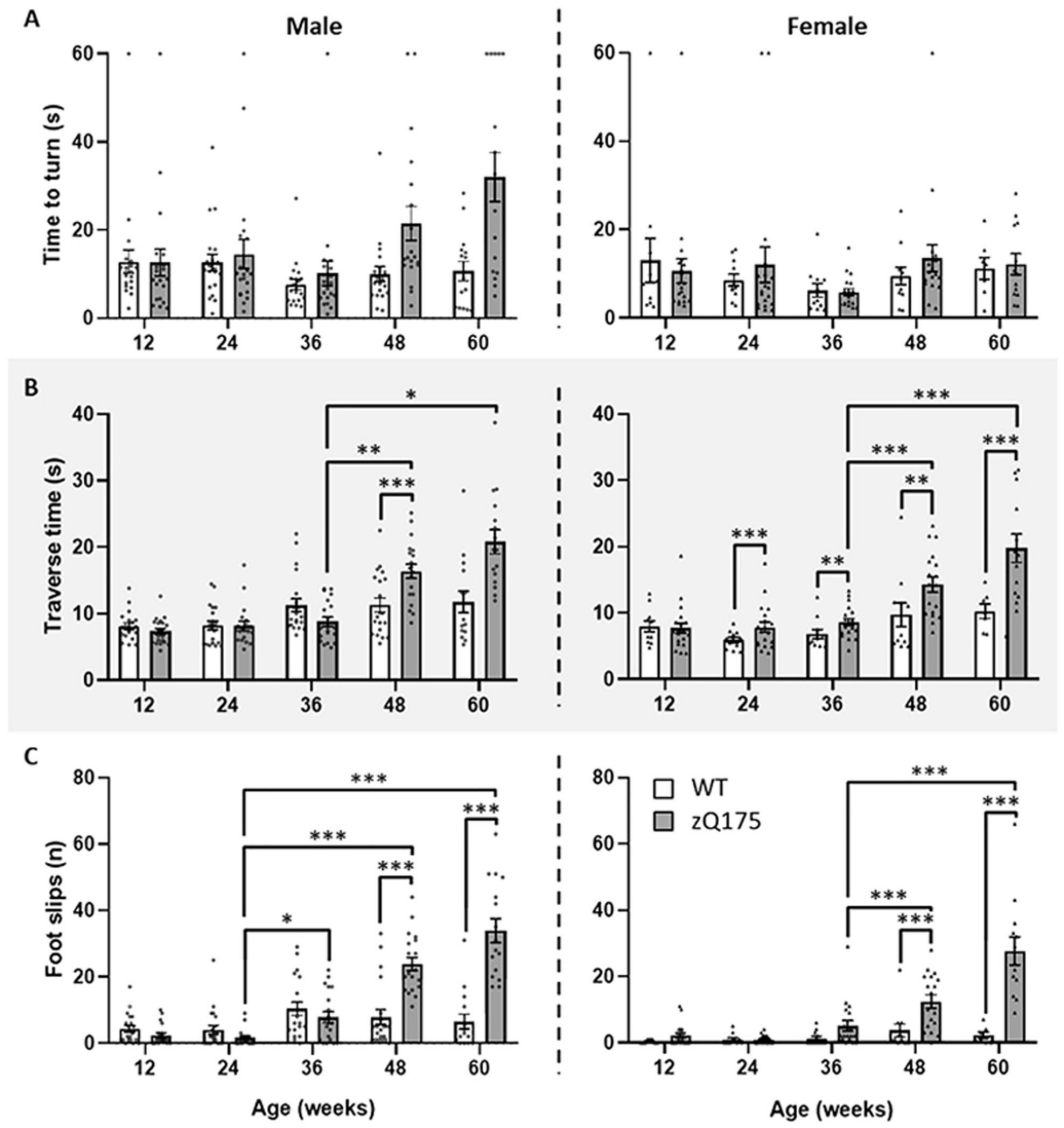
A) Time to turn on balance beam. Mean latency to turn 180° at the base of the beam for male and female mice (left and right panels respectively) from 12 to 60 weeks of age. B) Traverse time on balance beam. Mean time taken to cross the beam for male and female mice (left and right panels respectively) from 12 to 60 weeks of age. C) Foot slips from balance beam. Mean number of foot slips made whilst traversing the beam for male and female mice (left and right panels respectively) from 12 to 60 weeks of age. Data are presented as group means ± SEM; Pairwise comparisons: * *p* < .05, ** *p* < .01, *** *p* < .001.

The time to turn around, a measure of motor coordination on the balance beam task, saw no significant differences between zQ175 and WT animals for either males or females when analysed with weight as a covariate (Fig. 4A, Genotype: F_1,37_ = 3.91 and F_1,28_ = 2.09 respectively, both *n.s*.). Both male and female zQ175s took longer to cross the balance beam than sex-matched WT littermates (Fig. 4B, Genotype: F_1,37_ = 6.57, *p* < .05 and F_1,28_ = 24.18, *p* < .001 respectively) taking weight into account. For males, this difference was apparent only at 48 weeks of age (Genotype x Time: F_4,148_ = 3.33, *p* < .05; pairwise comparisons 48w, *p* < .001, all others *n.s*.). In contrast, female zQ175s were slower than WT from 24 weeks of age (Genotype x Time: F_4,112_ = 3.36, *p* < .05; pairwise comparisons 12w, *n.s*.; 24w, *p* < .001; 36w and 48w, both *p* < .01 and 60w, *p* < .001). Time to cross did not change over time for either male or female WTs (pairwise comparisons WT 12w vs 60w, both *n.s*.), whereas both male and female zQ175 performance significantly declined from 36 weeks (M zQ175 36w vs 48w, *p* < .01; 36w vs 60w, *p* < .05; F zQ175 36w vs 48w and 36w vs 60w, both *p* < .001). Total number of foot slips was increased in both zQ175 males and females compared to WT (Fig. 4C, Genotype: F_1,37_ = 25.19 and F_1,28_ = 34.72 respectively, both *p* < .001) when analysed including weight as a covariate. In both male and females zQ175 performed worse than WT from age 48 weeks onwards (Genotype x Time: F_4,148_ = 8.67 and F_4,112_ = 15.58 respectively, both *p* < .001; pairwise comparisons 12w – 36w, *n.s*.; thereafter, all *p* < .001).

The performance of male zQ175 declined from 24 weeks (M zQ175 24w vs 36w, *p* < .05; 24w vs 48w and 24w vs 60w, *p* < .001), and female zQ175 from 36 weeks (F zQ175 36w vs 48w and 36w vs 60w, both *p* < .001). In contrast, no decline in WT performance was detected (WT 12w vs 60w, M and F both *n.s*.).

### 3.7 Pavlovian classical conditioning task

**Fig. 5:**
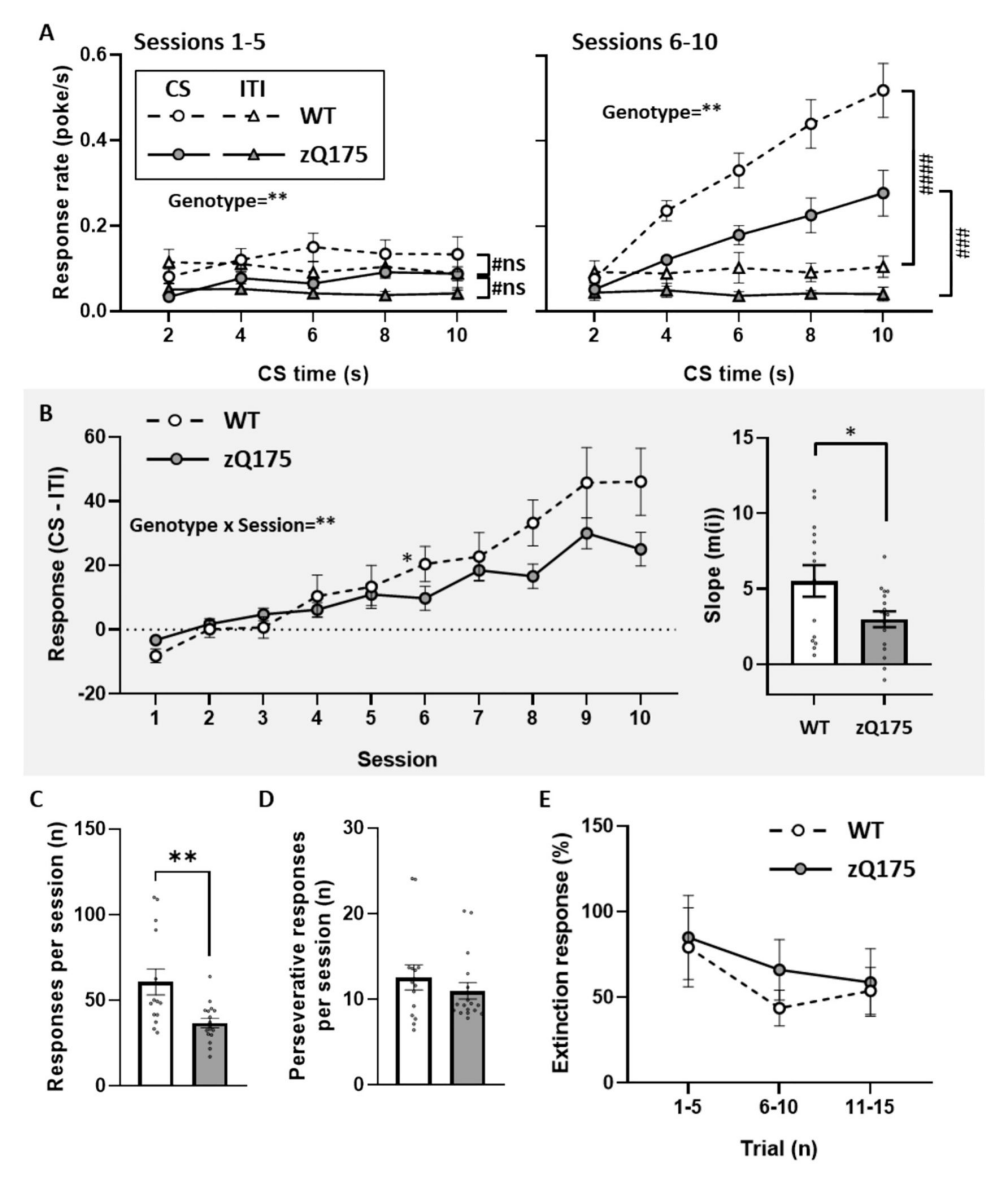
A) Classical conditioning: response rate. Mean number of responses made during the 10 s CS and ITI periods in 2 s time-bins within sessions 1–5 (left panel) and 6–10 (right panel). B) Classical conditioning: net CS response. Mean number of responses made in the 10 s CS minus background ITI responses per session (left panel) and learning slope over the ten sessions (right panel). C) Classical conditioning: total responses. Mean number of responses made in each session. D) Classical conditioning: total perseverance responses. Mean number of responses made in each session following collection of the reward. E) Classical conditioning: extinction test. Mean net CS responses by trial as a percentage of net CS responses of final rewarded session (session 10). From 12 weeks of age. Data are presented as group means ± SEM; Pairwise comparisons: Genotype effects: * *p* < .05, ** *p* < .01, *** *p* < .001; Stimulus effects: # *p* < .05, ## p < .01, ### *p* < .001.

Pavlovian learning was probed using a classical conditioning task during which the number of responses made to the reward magazine were recorded during a 10 s conditioned stimulus (CS) and the final 10 s of the preceding inter-trial interval (ITI). Over the first five sessions, zQ175 animals made fewer responses into the reward magazine on average, compared to WT (Fig. 5A **left panel**, Genotype: F_1,29_ = 10.40, *p* < .01), and there was no significant difference in responses between ITI and CS for either group (pairwise comparisons both *n.s*.). Throughout the final five sessions, zQ175 mice continued to respond at a lower rate than WT mice (Fig. 5A **right panel**, Genotype: F_1,29_ = 8.67, *p* < .01), but both zQ175s and WT learned to associate the CS with the reward (CS vs ITI, both *p* < .001). As the 10 sessions progressed, net CS responses of WT mice increased beyond that of zQ175 (Fig. 5B **left panel**, Genotype x Session: F_9,261_ = 2.64, *p* < .01) and zQ175 mice increased CS responding at a slower rate (Fig. 5B **right panel**, *t*_29_ 2.31, *p* < .05). The total number of magazine responses per session was also lower in the zQ175 compared to WT (Fig. 5C, *t*_29_ = 3.21, *p* < .01), however the number of perseverative responses in the magazine after reward had been collected was equal across groups (Fig. 5D, *t*_29_ = 0.91, *n.s*.). There was no significant difference in the extinction response between zQ175 and WT animals (Fig. 5E, Genotype: F_1,29_ = 0.35, *n.s*.), suggesting a similar capacity to inhibit previously acquired learning in both genotypes.

### 3.8 Progressive ratio task

**Fig. 6:**
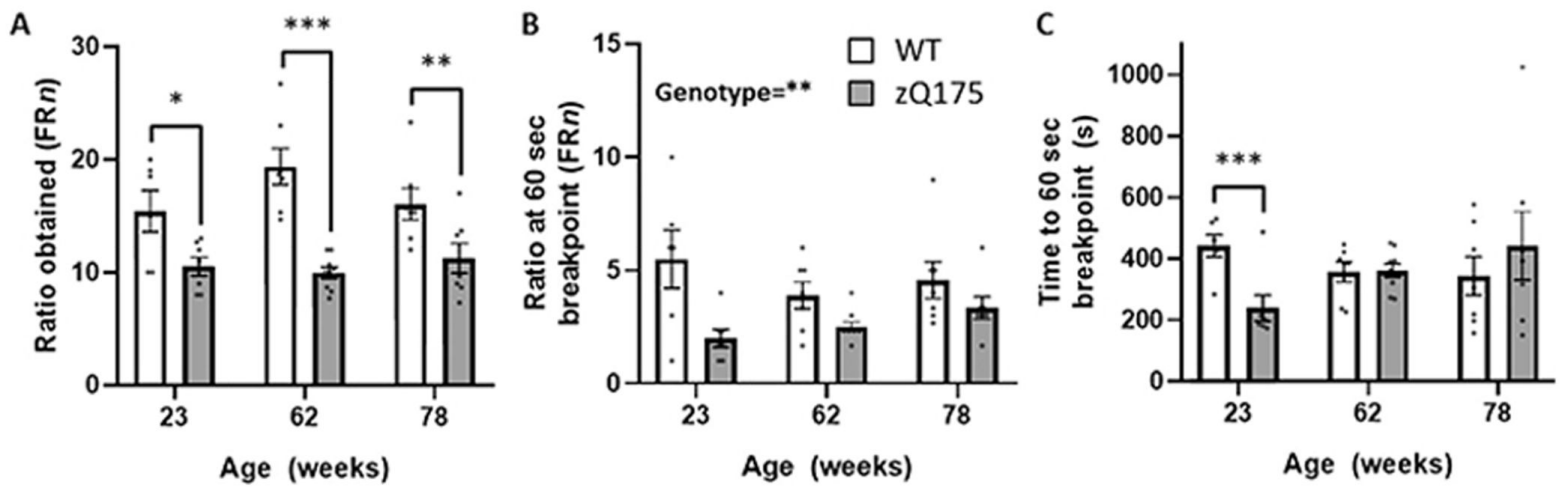
A) Progressive ratio: ratio obtained. Mean fixed ratio (FR) obtained, where n = number of responses required to obtain a reward. B) Progressive ratio: ratio at 60 s breakpoint. Mean ratio obtained before a break of 60 s was take. C) Progressive ratio: time to 60 s breakpoint. Mean time before a 60 s break was taken. 23 to 78 weeks of age. Data are presented as group means ± SEM; Pairwise comparisons at each age: * *p* < .05, ** *p* < .01, *** *p* < .001.

In a progressive ratio test used to probe the motivation to work for a reward, WT mice consistently reached a higher response-to-reward ratio than zQ175s from 23 weeks onwards (Fig. 6A, Genotype x Age: F_2,28_ = 5.49, *p* < .01; pairwise comparisons 23w, *p* < .05; thereafter, all *p* < .01). The ratio obtained by the time mice took a 60 s break before responding again (breakpoint) was significantly lower for zQ175 than WT (Fig. 6B, Genotype: F_1,14_ = 13.79, *p* < .01). At 23 weeks the WT mice worked for twice as long as zQ175 before taking a break of 60 s, however there was no significant difference from 62 weeks onwards (Fig. 6C, Genotype x Age: F_2,28_ = 4.48, *p* < .05; pairwise comparisons 23w, *p* < .001; there-after, all *n.s*.).

### 3.9 5 choice serial reaction time task

**Fig. 7:**
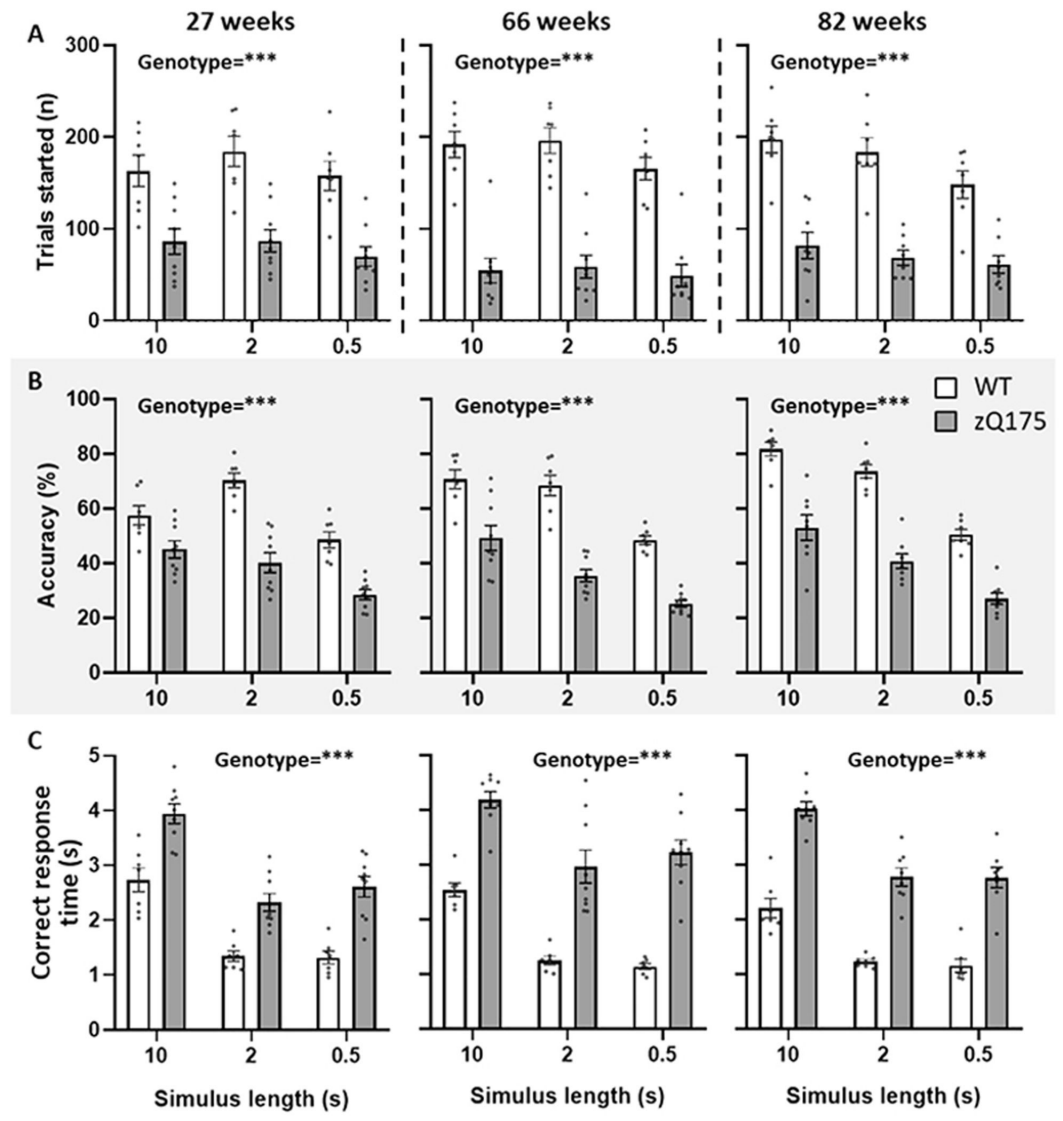
A) 5CSRTT: total trials started. Mean number of trials for which a response was made for each stimulus duration in the five-choice serial reaction time task. B) 5CSRTT: accuracy. Mean accuracy calculated as correct / correct + incorrect trials for each stimulus duration. C) 5CSRTT: correct response time. Mean time taken to make a correct response following stimulus presentation. 27 to 82 weeks of age. Data are presented as group means ± SEM; Pairwise comparisons: * *p* < .05, ** *p* < .01, *** *p* < .001.

The 5CSRTT measures visuospatial function, attention and impulsivity. zQ175 mice initiated fewer trials than WT at all ages (Fig. 7A, Genotype 27w, F_1,14_ = 21.31; 66w, F_1,14_ = 51.78 and 82w, F_1,14_ = 45.34, all *p* < .001). This deficit was consistent between the easiest (10 s), intermediate (2 s) and most difficult (0.5 s) stimulus durations. For those trials that were successfully initiated, zQ175 mice were less accurate than WT at responding correctly at all ages and levels of task difficulty (Fig. 7B, Genotype 27w, F_1,14_ = 28.35; 66w, F_1,14_ = 55.24 and 82w, F_1,14_ = 70.37, all *p* < .001), with no significant difference in performance between easy, intermediate and difficult stimulus durations. For those trials that were initiated and in which a correct response was made, zQ175 took longer to respond than WT at all time points (Fig. 7C, Genotype 27w, F_1,14_ = 31.32; 66w, F_1,14_ = 78.69 and 82w, F_1,14_ = 101.11, all p < .001), regardless of stimulus duration.

**Fig. 8:**
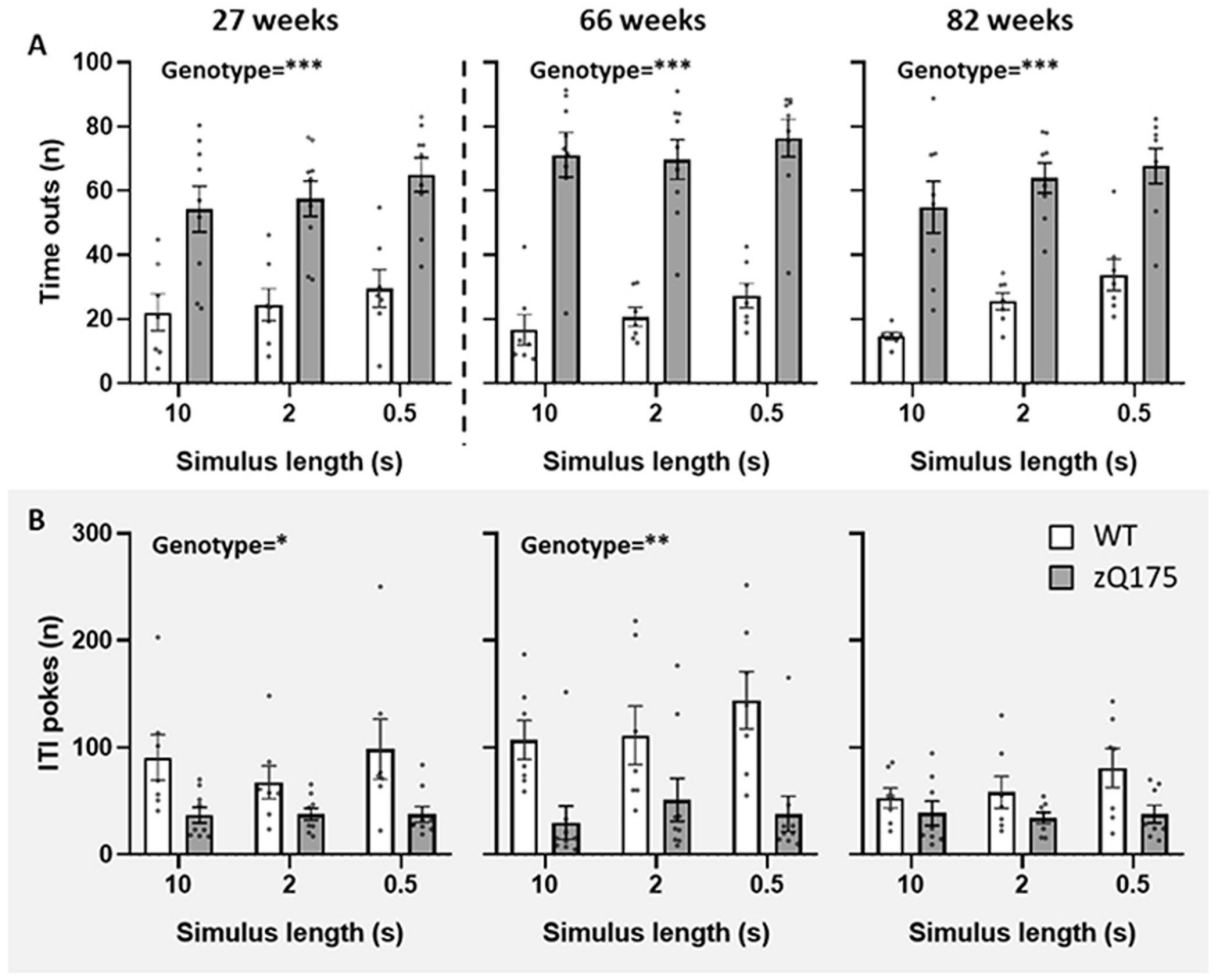
A) 5CSRTT: total time outs. Mean number of trials for which no response was made. B) 5CSRTT: total ITI pokes. Mean number of responses made during the inter-trial intervals. 27 to 82 weeks of age. Data are presented as group means ± SEM; Pairwise comparisons: * *p* < .05, ** *p* < .01, *** *p* < .001.

The number of stimuli for which no response was made was also greater for zQ175s across all time points (Fig. 8A, Genotype 27w, F_1,14_ = 18.38; 66w, F_1,14_ = 45.28 and 82w: F_1,14_ = 36.33, all *p* < .001) and all stimulus lengths. The number of responses made during the five-second inter-trial interval (ITI), taken as a measure of impulsivity, was lower for zQ175 than for WT at 27 weeks and 66 weeks (Fig. 8B, Genotype: F_1,14_ = 6.16, *p* < .05 and F_1,14_ = 9.67, *p* < .01 respectively). By 82 weeks of age, WT ITI responses had reduced to zQ175 levels (Genotype: F_1,14_ = 3.89, *n.s*.).

### 3.10 Serial implicit learning task

**Fig. 9:**
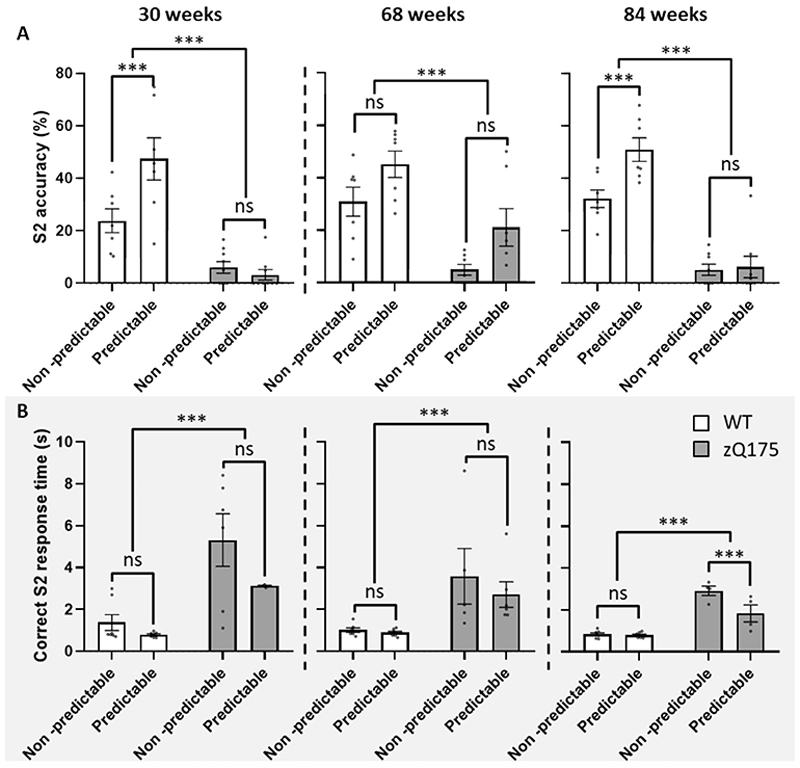
A) SILT: S2 accuracy. Mean accuracy to second stimulus during the randomized and predictable trials. B) SILT: correct S2 response time. Mean time taken when making a correct response to the second stimulus. 30 to 84 weeks of age. Data are presented as group means ± SEM; Pairwise comparisons: * *p* < .05, ** p < .01, *** *p* < .001.

This task requires the mouse to make a sequence of responses to obtain a reward. The majority of sequences are unpredictable and require explicit attention to be focused on the stimuli that are presented, while one predictable sequence is embedded in the task, allowing us to probe whether implicit learning is affected in this model. The number of initiated trials remained low in zQ175 compared to WT throughout the serial implicit learning task (Supplemental fig. 1, Genotype: F_1,14_ = 86.60, *p* < .001). Furthermore, of the trials completed, zQ175 were less accurate in responding in the second stimulus (S2) at all time points compared to WT (Fig. 9A, Genotype 30w, F_1,14_ = 36.03; 68w, F_1,14_ = 34.0 and 84w, F_1,14_ = 94.77, all *p* < .001). At 30 weeks, WT mice responded more accurately when presented with the predictable 2-step sequence compared to similar random non-predictable sequences, whereas no effect was observed in zQ175 performance (Genotype x Paradigm: F_1,14_ = 15.21, *p* < .01; pairwise comparisons WT, *p* < .001 and zQ175, *n.s*.). By 68 weeks, no significant difference in WT or zQ175 performance in either paradigm was detected (Genotype x Paradigm: F_1,14_ = 0.02, *n.s*.). At age 84 weeks, WT mice performed better in predictable trials compared to non-predictable in contrast to zQ175 for which no significant difference was observed (Genotype x Paradigm: F_1,14_ = 9.37, *p* < .05; pairwise comparisons WT, *p* < .001 and zQ175, *n. s*.). The time taken to make a correct response to S2 was greater for zQ175 than WT at all time points (Fig. 9B, Genotype 30w: F_1,14_ = 37.10, *p* = 2.8 × 10^−5^; 68w, F_1,14_ = 20.80 and 84w, F_1,14_ = 240.73, all *p* < .001). No significant difference in the speed to which WT and zQ175 responded in predictable trials compared to non-predictable was seen at either 30 or 68 weeks (Genotype x Paradigm: F_1,14_ = 2.16 and F_1,14_ = 2.63 respectively, both *n.s*.). However, by age 84 weeks, zQ175 mice were responding correctly in a faster time to predictable trials compared to non-predictable, whilst WT were equally fast in both paradigms (Genotype x Paradigm: F_1,14_ = 9.55, *p* < .01; pairwise comparisons WT, *n.s*. and zQ175, *p* < .001).

### 3.11 Delayed alternation

**Fig. 10:**
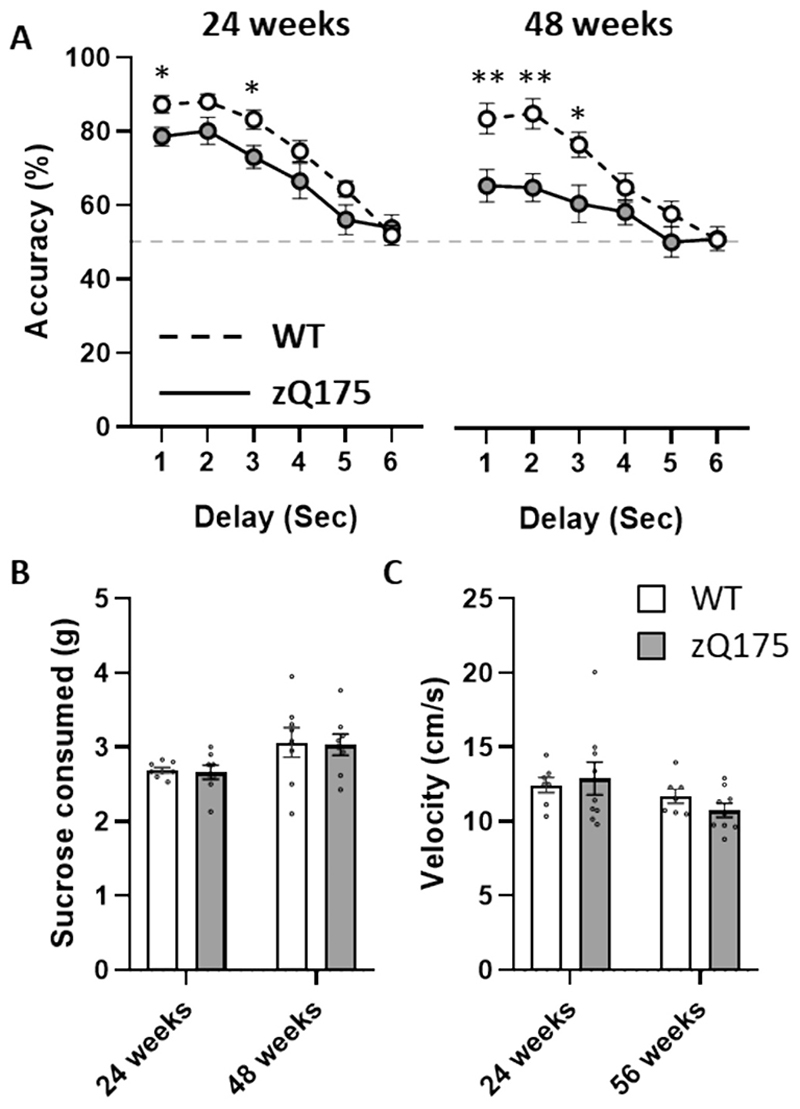
A) Delayed alternation. Mean accuracy calculated as correct / correct + incorrect trials after each delay duration at 24 and 48 weeks of age (left and right panels respectively). B) Sucrose consumption test. Mean weight of sucrose pellets consumed in the consumption test at 24 and 48 weeks of age. C) Locomotor velocity. Mean velocity as measured in an open field arena at 24 and 56 weeks of age. Data are presented as group means ± SEM; Pairwise comparisons: * p < .05, ** p < .01, *** *p* < .001.

A delayed alternation task was used to probe spatial working memory at 24 and 48 weeks old. During shorter delay trials (easier), zQ175 responded less accurately than WT (Fig. 10A, Genotype x Age x Delay: F_5,70_ = 2.57, *p* < .05; pairwise comparisons at 24w 1 s, *p* < .05; 2 s, *n.s*.; 3 s, *p* < .05; 48w 1 s and 2 s, both *p* < .01; 3 s, *p* > .05).zQ175, but not WT, performance declined between testing points (WT 24w vs 48w, *n.s*. and zQ175 24w vs 48w, *p* < .01). For the longer delay trials (harder), WT accuracy dropped to zQ175 levels (24w and 48w 4–6 s, all *n.s*.).

### 3.12 Sucrose consumption test

The amount of sucrose reward consumed when given free access did not differ significantly between zQ175 and WT at either 24 or 48 weeks of age (Fig. 10B, Genotype: F_1,14_ = 0.09, *n.s*.).

### 3.13 Velocity

Mean locomotor velocity was measured in an open field arena at 24 and 58 weeks of age. No significant differences between zQ175 and WT were observed (Fig. 10C, Genotype: F_1,14_ = 0.14, *n.s*.).

### 3.14 Summary

**Fig. 11:**
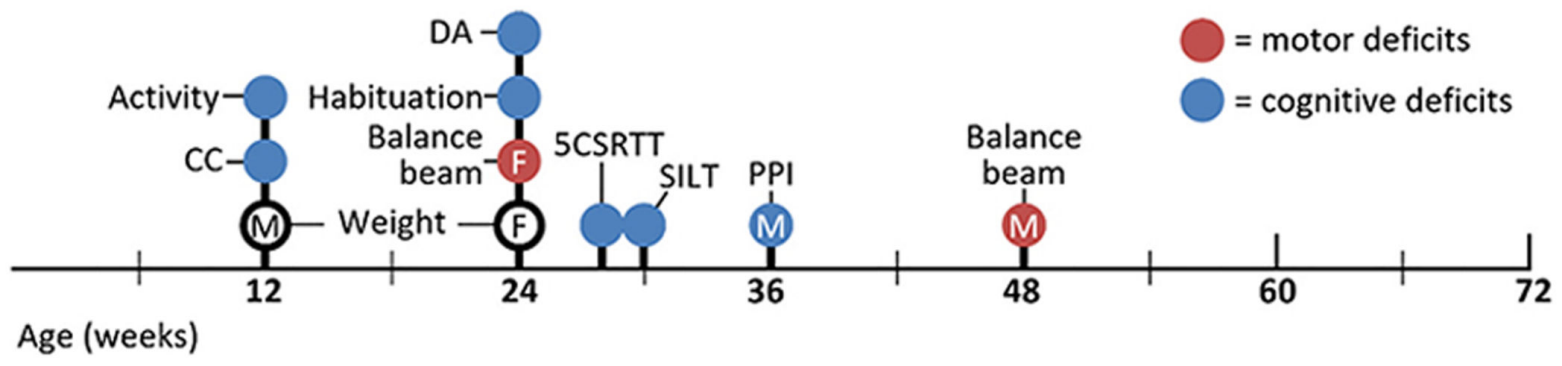
Timeline summarizing the manifestation of behavioural deficits identified by different tests in zQ175 mice. Red circles represent motor deficit, blue circles represent cognitive deficits. M = male only, F = female only; CC = classical conditioning test, DA = delayed alternation test, 5CSRTT = five-choice serial reaction time task, SILT = serial implicit learning task, PPI = pre-pulse inhibition. (For interpretation of the references to colour in this figure legend, the reader is referred to the web version of this article.)

A schematic depicting the timeline of key cognitive and neuropsychiatric changes is presented in Fig. 11, with a summary of significant changes detailed in Table 1.

### 3.15 Histology

**Fig. 12:**
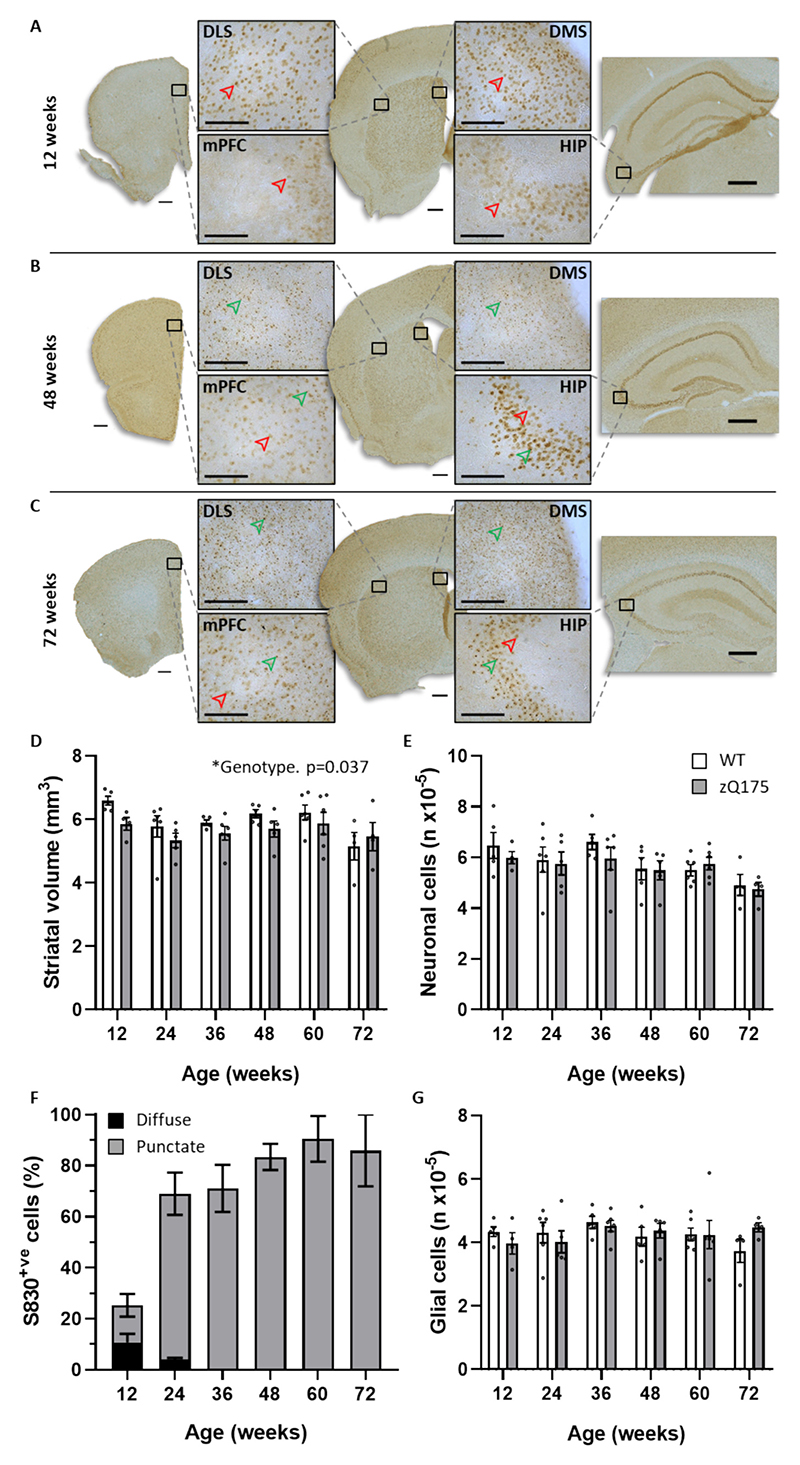
Histological analysis of the zQ175 brain tissue between 12 and 72 weeks of age. Representative immunohistochemical analysis of mHTT using S830 antibody in the mPFC, DLS, DMS and HIP brain regions at 12 (A), 48 (B) and 72 (C) weeks of age in zQ175 mice. (D) Striatal volume (mm^3^) of WT and zQ175 mice. (E) Mean neuronal cell counts in the striatum of WT and zQ175 mice. (F) Mean number of cells positive for S830 mHTT labelling, either as diffuse only (black bars) or with punctate staining (grey bars) in zQ175 mice. (G) Mean number of glial cells in the striatum of WT and zQ175 mice. Error bars = ±SEM Individual dots represent data points from individual mice. White bars = WT mice; Grey bars = zQ175 mice. Scale bars on macro images = 400 μm and on inserts = 100 μm. Red arrows indicate diffuse stained cells. Green arrows indicate inclusions. mPFC = medial prefrontal cortex; DLS = lateral portion of the dorsal striatum; DMS = medial portion of the dorsal striatum; HIP = hippocampus. (For interpretation of the references to colour in this figure legend, the reader is referred to the web version of this article.)

Staining for mHTT was undertaken using S830 antibody (Fig. 12, A-C) and pathology is evident as early as 12 weeks. Diffuse staining is predominately evident at 12 weeks of age, while more punctate aggregates are evident at the 48 and 72-week timepoints. Volumetric analysis of the brain revealed that zQ175 mice presented with a reduced striatal volume overall [Fig. 12D, Genotype: F_1,49_ = 4.61, *p* = .037]. No difference in the number of neuronal cells [Fig. 12E, Genotype: F_1,49_ = 0.84, *p* = .364], at any age was observed. Stereological quantification of the staining revealed punctate staining as early as 12 weeks, the amount of this staining increased drastically from 12 to 24 weeks [Fig. 12F, F_5,34_ = 9.35, *p*.002] where it remained constant until 72 weeks [Fig. 12F, minimum *p* value from post hoc comparisons: F_5,34_ = 9.35, *p* = .30]. Diffuse staining was evident at 12 and 24 weeks, but dramatically reduced by 36 weeks of age [Fig. 12F, F_5,34_ = 11.94, *p* < .001]. No differences in glial cells were evident at any age [Fig. 12G, Genotype: F_1,49_ = 0.19, *p* = .891].

## 4 Discussion

Understanding complex neurodegenerative diseases necessitates the use of preclinical models, and mouse models are particularly well suited to elucidate the role of gene mutations in disease pathophysiology and behaviour. Here, we ask how a gene construct relevant to Huntington’s disease results in the progressive manifestation of cognitive, motor and neuropsychiatric changes, by using a battery of tests that measure novelty response, attention, impulsivity, classical conditioning, implicit learning, sensorimotor gating, extinction learning, memory, motivation and a range of motor behaviours, between 12 and 84 weeks of age. The test battery was chosen to represent a range of HD symptoms and prodromal signs to characterise the emergence of these complex behavioural changes. These data allow us to establish a phenotypic timeline to aid appropriate model selection in which future interventions may be tested.

### 4.1 zQ175 mice weigh less than WT

We show that zQ175 mice weigh less from an early age and fail to catch up with WT growth, a feature exhibited earlier and more pronounced in males. The prodromal body mass index of people with HD is consistently lower compared to age and sex-matched control groups, with evidence suggesting males are more affected than females (Costa de Miranda et al., 2019; Djoussé et al., 2002). This phenotype emerges well before any motor symptoms and is thought to be linked to metabolic deficits caused by mHTT (Aziz and Roos, 2013). As a result of this, our motor tests known to be influenced by body weight were analysed with weight as a covariant.

### 4.2 Fine motor but no gross motor deficits from mid-age in zQ175 mice

Few motor deficits were detected in our model, and those that were identified emerged during the later time-points. No significant deficits were seen in two of the most used tests of gross motor function (rotarod and grip strength) even up to 60 weeks of age, consistent with previous findings (Southwell et al., 2016). However, the more sensitive balance beam test showed loss of fine balance control in zQ175 mice from 48 weeks old, and deficits in females specifically from as early as 24 weeks of age in the traverse time outcome measure. Previous studies using only male mice also reported balance deficits from 12 months (Loh et al., 2013), and human studies identified earlier deficits in females (Zielonka et al., 2013). These results demonstrate slow motor deficit progression, reflective of that seen in people with HD. More so than for more severe late-stage disease models such as R6/1, the zQ175 model presents an opportunity for testing long-term treatments and their effects on fine motor impairments. zQ175 could be a useful preclinical model, particularly for non-pharmacological interventions that may have a subtle yet cumulative effect over time such as the effect of environment, diet or exercise on later-life outcome. In addition, the relatively slow progression of motor signs could enable administration of cell transplantation therapies after the manifestation of first motoric disease signs with sufficient time for grafts to integrate into the host parenchyma to demonstrate functional benefits (Cisbani et al., 2014; Dunnett et al., 1998). This becomes harder with shorter-lived or later-stage models where disease phenotypes may have advanced too far to benefit from the treatment.

### 4.3 Variable activity differences in zQ175

Male zQ175s explored less than WT after being placed in activity monitoring cages and were slower to habituate from 24 weeks onwards. At the earliest time-points they moved around less than WT in both the light and dark periods, but this difference was not maintained as they aged. In fact, by 60 weeks the zQ175s were moving around more than the WT at night. Female zQ175 pattern of activity as they aged was more variable, showing activity deficits only at 24 weeks, but increased daytime activity at 36 weeks. As with males, the female zQ175s were hyperactive compared to WT at night. Differences in habituation activity between genotypes in females did not reach significance. Given that zQ175s of both sexes were capable of moving around more than they did during the daytime (they moved four times as much in the same time-frame during the dark period), and the fact that no deficits in motor function tests or velocity were seen, suggests that the early appearance of reduced locomotor activity may be a result of non-motor changes. For example, body temperature and metabolic changes have been linked to changes in activity levels (Hankenson et al., 2018; Mayer et al., 1990), disturbances which have previously been identified in the zQ175 model (Nateghi et al., 2024; Tanghe et al., 2021), and indeed are known to be disrupted for people with HD (Collazo et al., 2022; Singh and Agrawal, 2021). Although psychiatric conditions cannot readily be extrapolated to animal models, apathy could also be an explanation for early motoric reductions, as we observed deficits in motivation in the zQ175s, which is modulated by common neural circuits (Steffens et al., 2022), and fluctuating activity levels in people can result from low mood, another common feature of HD (Gianfredi et al., 2022; Peyser and Folstein, 1990).

### 4.4 Reduced ability to inhibit startle response in zQ175 males

Their ability to inhibit their startle response with a pre-pulse warning declined over time in both male and female zQ175 mice; however, only the males were significantly impaired compared to their sex-match WT littermates. From age 36 weeks onwards, the males demonstrated an inability to suppress their response, suggesting a deficit in sensorimotor gating. This impairment in Q175 mice is consistent with deficits in human HD, whereby both male and female patients presented with an impaired pre-pulse inhibition response to both acoustic and tactile stimuli (Beste et al., 2011; Soloveva et al., 2020; Swerdlow et al., 1995).

### 4.5 Learning and motivational deficits but no impulsivity in younger zQ175

Sensitive operant-based tests can detect subtle changes in executive function driven by changes within cortical and basal ganglia systems. In contrast to motor function, the zQ175s showed significant cognitive deficits from 12 weeks old, the earliest time-point tested here. The zQ175 mice were able to learn stimulus-outcome associations, as demonstrated in the classical conditioning (Pavlovian learning) task in which response rate increased when a conditioned light stimulus (CS) linked to reward delivery was presented. They also showed cognitive flexibility by extinguishing this association at the same rate as WT when the CS was disassociated from the reward in an extinction test. However, their peak CS response rate in rewarded trials was 50 % of WT and their rate of learning across sessions was slower. Learning deficits have been linked to increased apathy and impulsiveness in prodromal HD (McLauchlan et al., 2019; Morris et al., 2022, 2024), both of which were measured in our test battery. The impulsivity measure of the 5CSRTT (number of hole pokes made during the ITI before the presentation of the stimulus (Asinof and Paine, 2014)) did not indicate any increase in impulsivity traits in the zQ175s; in fact, this was reduced. Since apathy is a human trait, this cannot directly translate to mice, however we can measure motivation by the effort they will expend to gain a reward as a proxy. General engagement of zQ175 mice with the reward magazine during the CCT was reduced in comparison to WT. This trend was obvious at the earliest timepoints for every operant task, with zQ175 initiating significantly fewer trials and failing to respond more frequently in the 5CSRTT, SILT and delayed alternation tasks, even during the ‘easiest’ versions (i.e. longest stimulus durations). Similar low response rates have been identified previously in touch screen operant tasks (Piiponniemi et al., 2018). The progressive ratio data confirms a reduced motivation in the zQ175s, with WT mice working harder for the same amount of reward, attaining a higher response per reward ratio. Furthermore, zQ175 would break from responding sooner and at a lower response:reward ratio compared to WT. Although people with pre-manifest HD are sensitive to the perceived value of a reward, it has been reported that they are insensitive to the loss of reward and do not alter their behaviour to adapt to reward loss (Enzi et al., 2012), a trait that may contribute to low response rates. Our consumption test showed that when the reward was made freely available there was no difference in the amount consumed between the zQ175 and WT mice, indicating that the response deficit isn’t a result of disinterest in or devaluation of the reward itself, but perhaps a reduction in the perceived value of the reward when balanced with the effort required to obtain it.

### 4.6 Attentional and visuospatial response deficits in younger zQ175

Despite low response rates, zQ175 mice were able to learn to respond to the correct stimulus in 5CSRTT. Of the trials completed, they responded correctly around 45 % of the time - more accurately than by chance alone (20 %). Introducing the attentional component of the task (reduction of stimulus duration), reduced zQ175 accuracy, indicative of an attentional deficit. However, even during the longest stimulus duration when attention was less critical, their accuracy was still significantly lower than that seen in the WT mice. In the case of incorrect responses, the mice had made the effort to respond, they were just inaccurate, which may suggest that performance is also affected by a visuospatial response deficit, since people with HD can experience visuospatial discrepancies contributing towards similar instrumental learning deficits (Labuschagne et al., 2016; Lawrence et al., 2000). Learning in HD has also been shown to be differentially affected by positive or negative outcomes to responses (McLauchlan et al., 2019; Morris et al., 2022), suggesting that zQ175 learning may be disrupted due to reduced learning from lack of reward when incorrect responses are made, rather than as a result of impaired learning from rewarded actions.

The time taken to respond correctly to a stimulus was consistently longer for zQ175 in the 5CSRRT and SILT tasks. This is also likely to be driven by an attentional and/or motivational deficit, rather than a motor impediment, since the increased response latency was evident before any motor changes were identified. Furthermore, when left to freely explore the open field arena, we found no difference in the velocity of locomotion between zQ175 and WT mice, although further analysis of the open field data divided into centre versus outer arena would be useful to validate this pattern. When responding to a predicable stimulus, it was shown that zQ175 were able to action a faster response when less thought processing was required in making that response, albeit at an older age. Correct responses made to the predictable stimulus are driven less by attention and more by implicit muscle memory.

### 4.7 Deficits in implicit learning but intact working memory in zQ175 mice

Measuring the accuracy of S2 responses in the SILT task showed that implicit learning in zQ175 was disrupted from 30 weeks old. In contrast to the WT mice, accuracy in responding to a predictable stimulus was not increased in comparison to those presented at random, a deficit common in premanifest HD patients (Ghilardi et al., 2008).

The delayed alternation task challenges the working memory performance of mice by requiring them to hold information about the task in their working memory for up to 6 s before being able to provide a response in the task. Wildtype mice perform well at short delays, but accuracy declines as the working memory demands increase up to 6 s, where they eventually only respond at chance (50 %). An impairment in working memory would result in reduced accuracy as the task demands (i.e. delays) increase, beyond the normal decline observed in wildtype mice. Interestingly, however, zQ175 mice present with an impairment at the shorter delays, and their accuracy levels do not decline more rapidly than wildtype mice at the longer delays. This suggests that the zQ175 mice may have an impairment in alternation behaviour or rule learning, but with working memory capacity similar to wildtype mice. Interestingly, this stands in contrast to the early emergence of spatial working memory deficits reported in people with HD, where (Glikmann-Johnston et al., 2019; Lemiere et al., 2002) both pre-manifest and early symptomatic HD patients present with impaired working memory (Glikmann-Johnston et al., 2019; Harris et al., 2019; Lawrence et al., 1996). While it is challenging within this work to explain this discrepancy between the rodent model and human data, it is well documented that spatial working memory deficits in delayed alternation tasks are associated with hippocampal, prefrontal and retrosplenial cortex function (Aggleton et al., 1986; Bizon et al., 2012; Subramanian et al., 2024). Further investigation of human HD versus the zQ175 model may shed some light on discrepancies in the pattern or extent of pathology within these regions. A final consideration is that our last experimental assessment on the delayed alternation task was conducted at 48 weeks of age, so later assessment is warranted to determine whether this feature manifests at a later stage in the zQ175 model.

### 4.8 Pathology

The zQ175 model has been characterised histologically previously, with labs reporting evidence of mHTT expression as early as 6–8 weeks of age (Smith et al., 2023). In line with this, we observe mHTT at the earliest time point assessed, 12 weeks of age, as well as the anticipated change from early diffuse staining to later punctate aggregates. Volumetric analysis of the striatum revealed reduced striatal volume in our histology, which is consistent with brain atrophy that has been reported in this model previously (Heikkinen et al., 2012; Peng et al., 2016). Our analysis also did not reveal evidence of neuronal or glial cell loss, which is consistent with some reports that NeuN protein levels are not altered in the striatum of heterozygous zQ175 mice at 12 months of age (Peng et al., 2016). The same laboratory did, however, report reduced striatal DARPP-32 at this age, suggesting neuronal pathology may be a key driver of functional changes. Consistent with this, our lab have previously undertaken Golgi-cox staining of medium spiny neurons in 12-month old zQ175 and demonstrated similar numbers of dendritic branches and similar complexity of these dendrites, but fewer spines per μm in the HD mice (Bayram-Weston et al., 2016b), providing further evidence of neuronal dysfunction in this model.

### 4.9 Sex differences

While a strength of this study was the inclusion of a well-powered cohort (Cohort 1), allowing statistical analysis of sex-differences on a range of tasks, the study was limitated by the inclusion of two smaller cohorts (Cohort 2, 3), which were pooled due to the lower number of mice per genotype. As we noted some sex differences in the onset or extent of behavioural dysfunction on the balance beam task, locomotor activity and pre-pulse inhibition tasks, this suggests that sex differences may be apparent across the behavioural battery more broadly. Indeed, as discussed in the section below, sex differences have also been observed in other genetic models of HD. Future studies using any model of HD should endeavour to not only include mice of both sexes, but to ensure that each sex is sufficiently well-powered to allow statistical consideration of the effect of sex on the study measures.

### 4.10 Assessment of cognitive and neuropsychiatric phenotypes in alternative genetic models

We have investigated motor, cognitive and neuropsychiatric features of disease in the zQ175 model in this project, and considered the relationship between behavioural phenotypes in mice with the human condition. However, it is worth noting that many of these features have been explored, and reported, in other genetic models of HD. Here we provide examples of some of these changes, with a focus on neuropsychiatric changes that have been reported to be sex dependent. For example, in one study, female R6/1 mice were immobile for longer in the forced swim test, less immobile in the tail-suspension test and had a significantly longer latency response in the novelty-suppressed feeding test, all of which measure aspects of depressive-like behaviour (Pang et al., 2009). Similarly, Renoir and colleagues report that female R6/1 mice exhibited a decreased preference for saccharin and impaired emotionality-related behaviours when assessed on the novelty-suppressed feeding test and the forced swim test (Renoir et al., 2011, 2012). Interestingly, female R6/1 mice were also more vulnerable to stress in a short-term memory paradigm than male R6/1 mice (Mo et al., 2013), although exposure to oral corticosterone treatment accelerated impaired performance of male R6/1 mice on the same short-term memory task (Mo et al., 2014).

In a different line, the HdhQ111 model, female mice developed a depressive-like phenotype in the forced swim test, whereas male mice presented with an increased anxiety-like phenotype in the open field (Orvoen et al., 2012). Depressive-like behaviour has also been reported in the YAC128 model, using the Porsolt forced swim test and the sucrose intake test of anhedonia, with sex differences having less infleunce in this model (Pouladi et al., 2009). An anxiety-like phenotype has been revealed in the R6/2 line and BACHD models using the light/dark test, while this phenotype was not evident in YAC128 or HdhQ111 mice (L. Menalled et al., 2009). The pre-pulse inhibition test identified sensory-gating deficits in the R6/2 mice and BACHD mice, but not YAC128 and HdhQ111 models (L. Menalled et al., 2009). Impaired sociability was particularly evident in male R6/1 mice using the two-stimulus compartment test where they showed lower levels of affiliative behaviours in the social interaction test, although no differences in pre-pulse inhibition were evident in either sex in this study (Pietropaolo et al., 2011). Thus, although our data provide specific information on the zQ175 model, many important cognitive and neuropsychiatric features of disease are evident in alternative genetic models of disease.

### 4.11 Conclusion

The zQ175 mouse model of HD has high face-validity given the native CAG knock-in within the Htt exon. The resulting phenotypes offer progressive and translational readouts which clearly identify both motor and cognitive deficits. As with many people with HD, these cognitive deficits can be identified much earlier in the life of the mice compared to motor deficits (Fell and Gupta, 2023), which are subtle but detectable while general health and condition are still good. This slow progression of motor symptoms offers a relatively large window for conducting long-term pre-manifest interventions and assessing functional outcomes. A broader array of executive function impairments in zQ175 mice can be detected by simple but sensitive operant testing from a relatively young age, see summary Fig. 11. Proxy measures of many prodromal cognitive signs seen in people with HD can be identified. From the test batteries described here we were able to show reduced motivation, deficiencies in implicit learning and slower learning rates, attentional deficits, reduced startle response inhibition as well as dysfunction of visuospatial processing and motivation to work for reward, see Table 1. Interestingly, zQ175 mice do not present with the impaired working memory deficits observed in people with HD. Whilst motivation to engage in operant tasks may be lower than wildtype mice, even in young mice, zQ175s are still able to perform the tasks and learn complex rules. This provides scope for within-subject assessment of pharmaceutical or non-pharmaceutical therapies, since mice can be trained and assessed in multiple outcome measures for cognitive improvements.

## Supplementary Material

**Appendix A.Supplementary data** Supplementary data to this article can be found online at https://doi.org/10.1016/j.expneurol.2025.115350.

Supplementary Data

## Figures and Tables

**Table 1:** Breakdown of significance for outcome measure comparisons for each test. 5CSRTT = five-choice serial reaction time task, SILT = serial implicit learning task, CS = conditioned stimulus, ITI = inter-trial interval, S2 = 2nd stimulus; ↓ = decrease, ↑ = increase; ns = not significant, *p < .05, **p < .01, ***p < .001.

Test		Outcome measure	Sex	Difference compared to sex-matched WT						
				12w	24w	36w	48w	60w	72w	84w
		Weight	*M* *F*	↓ *** ns	↓ *** ↓ *	↓ *** ↓ **	↓ *** ↓ ***	↓ *** ↓ ***	↓ *** ↓ ***	
Rotarod		Latency to fall	*M* *F*	ns ns	ns ns	ns ns	ns ns	ns ns		
Grip strength		Latency to fall	*M* *F*	ns ns	ns ns	ns ns	ns ns	ns ns		
Locomotor activity		Habituation activity	*M* *F*	ns ns	↓ * ns	↓ ** ns	↓ * ns	↓ * ns		
		Light phase activity	*M* *F*	↓ *** ns	↓ ** ↓ *	ns ↑ *	ns ns	ns ns		
		Dark phase activity	*M* *F*	↓ *** ns	↓ ** ↓ **	ns ns	ns ns	↑ ** ↑ **		
Pre-pulse inhibition		% inhibition	*M* *F*		ns ns	↓ *** ns	↓ *** ns	↓ *** ns		
Balance beam		Time to turn	*M* *F*	ns ns	ns ns	ns ns	ns ns	ns ns		
		Traverse time	*M* *F*	ns ns	ns ↑ ***	ns ↑ **	↑ *** ↑ **	ns ↑ ***		
		Foot slips	*M* *F*	ns ns	ns ns	ns ns	↑ *** ↑ ***	↑ *** ↑ ***		
Classical conditioning		CS Response Ratio obtained		↓ *	↓ *			↓ ***		↓ **
Progressive ratio		Ratio at breakpoint			↓ *			↓ *		ns
		Time to breakpoint			↓ ***			ns		ns
		Trials started			↓ ***			↓ ***		↓ ***
		Accuracy			↓ ***			↓ ***		↓ ***
5CSRTT		Response time			↑ ***			↑ ***		↑ ***
		Time outs			↑ ***			↑ ***		↑ ***
		ITI pokes			↓ *			↓ **		ns
		Trials started				↓ ***			↓ ***	↓ ***
		S2 accuracy				↓ ***			↓ ***	↓ ***
SILT		Predicable accuracy				↓			↓	↓
		S2 response time				↑ ***			↑ ***	↑ ***
Delayed alternation		Accuracy			↓ *		↓ **			
Reward consumption		Sucrose consumed			ns		ns			
Open field		Velocity			ns			ns		

## Data Availability

Data will be made available on request.
