# Supplementary material for: Complex cognitive and motivational deficits precede motor dysfunction in the zQ175 (190 CAG repeat) Huntington’s disease model
Source: Exp Neurol. Author manuscript; Available in PMC 2026 Jun 19. (PMC7619202; doi:10.1016/j.expneurol.2025.115350)
Supplement: Supplementary Data [file EMS214116-supplement-Supplementary_Data_.docx]

Supplemental Figure

**
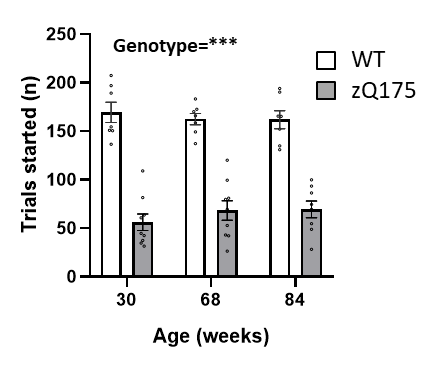
**

***Supplementary figure 1 SILT: total trials started.*** *Mean number of trials for which a correct response was made in the first stimulus (S1) from 30 to 84 weeks of age. Data are presented as group means ± SEM; Pairwise comparisons: * p<.05, ** p<.01, *** p<.001.*
